# Effect of Immediate Implant-Based Breast Reconstruction After Mastectomy With and Without Acellular Dermal Matrix Among Women With Breast Cancer: A Randomized Clinical Trial

**DOI:** 10.1001/jamanetworkopen.2021.27806

**Published:** 2021-10-01

**Authors:** Fredrik Lohmander, Jakob Lagergren, Hemming Johansson, Pankaj G. Roy, Yvonne Brandberg, Jan Frisell

**Affiliations:** 1 Section of Breast Surgery, Department of Breast and Endocrine Surgery, Karolinska University Hospital, Stockholm, Sweden; 2 Department of Molecular Medicine and Surgery, Karolinska Institutet, Stockholm, Sweden; 3 Department of Surgery, Breast Center, Capio St: Görans Hospital, Stockholm, Sweden; 4 Department of Oncology-Pathology, Cancer Center Karolinska, Karolinska Institutet, Stockholm, Sweden; 5 Department of Breast Surgery, Oxford University Hospitals NHS Foundation Trust, Oxford, United Kingdom

## Abstract

**Importance:**

The use of acellular dermal matrix (ADM) in implant-based breast reconstructions (IBBRs) is established practice. Existing evidence validating ADMs proposed advantages, including improved cosmetics and more single-stage IBBRs, is lacking.

**Objective:**

To evaluate whether IBBR with ADM results in fewer reoperations and increased health-related quality of life (HRQoL) compared with conventional IBBR without ADM.

**Design, Setting, and Participants:**

This was an open-label, multicenter, randomized clinical trial of women with primary breast cancer who planned for mastectomy and immediate IBBR, with a 2-year follow-up for all participants. Participants were enrolled at 5 breast cancer units in Sweden and the United Kingdom between 2014 and May 2017. Exclusion criteria included previous radiotherapy and neo-adjuvant chemotherapy. Data were analyzed until August 2017.

**Interventions:**

Participants were allocated to immediate IBBR with or without ADM.

**Main Outcomes and Measures:**

The primary trial end point was number of reoperations at 2 years. HRQoL, a secondary end point, was measured as patient-reported outcome measures using 3 instruments from the European Organization for Research and Treatment of Cancer Quality of life Questionnaire.

**Results:**

From start of enrollment on April 24, 2014, to close of trial on May 10, 2017, a total of 135 women were enrolled (mean [SD] age, 50.4 [9.5] years); 64 were assigned to have an IBBR procedure with ADM and 65 to the control group who had IBBR without ADM. There was no statistically significant difference between groups for the primary outcome. Of 129 patients analyzed at 2-year follow-up, 44 of 64 (69%) had at least 1 surgical event in the ADM group vs 43 of 65 (66%) in the control group. In the ADM group, 31 patients (48%) had at least 1 reoperation on the ipsilateral side vs 35 (54%) in the control group. The overall number of reoperations on the ipsilateral side were 42 and 43 respectively. Within the follow-up time of 24 months, 9 patients (14%) in the ADM group had the implant removed compared with 7 (11%) in the control group. We found no significant mean differences in postoperative patient-reported HRQoL domains, including perception of body image (mean difference, 3; 99% CI, −11 to 17; *P* = .57) and satisfaction with cosmetic outcome (mean difference, 8; 99% CI, −6 to 20; *P* = .11).

**Conclusions and Relevance:**

Immediate IBBR with ADM did not yield fewer reoperations compared with conventional IBBR without ADM, nor was IBBR with ADM superior in terms of HRQoL or patient-reported cosmetic outcomes. Patients treated for breast cancer contemplating ADM-supported IBBR should be informed about the lack of evidence validating ADM’s suggested benefits.

**Trial Registration:**

ClinicalTrials.gov Identifier: NCT02061527

## Key Points

**Question:**

Does acellular dermal matrix (ADM) in implant-based breast reconstruction reduce reoperation rates for women having immediate implant-based reconstruction in the setting of breast cancer treatment?

**Findings:**

In this randomized clinical trial comparing implant-based breast reconstruction with and without ADM among women with primary breast cancer, use of ADM in reconstruction did not yield fewer reoperations.

**Meaning:**

Women with breast cancer considering immediate implant-based breast reconstruction should anticipate several reoperations; clinics should inform patients about the limited evidence of benefit of ADM-assisted breast reconstruction.

## Teaser

This randomized clinical trial compares the amount of reoperations that occur in implant-based breast reconstructions using an acellular dermal matrix vs reconstructions without matrices.

## Introduction

Breast cancer is the most common form of cancer in women, annually affecting some 1.4 billion patients worldwide and accounting for 23% of all cancers.^1^ Novel oncoplastic techniques for breast conserving surgery have gained popularity, while many women still undergo mastectomy.^2^ Breast reconstruction following mastectomy can have health benefits and is considered a quality measure for breast cancer care in Sweden and the United Kingdom.^3^ Autologous and alloplastic reconstructive options are available, both with advantages and limitations.^4^ Implant-based breast reconstruction (IBBR) remains predominant, and acellular dermal matrix (ADM) is commonly used in these procedures.^5^ ADM as a device for breast reconstruction aims to combine the advantages of early subcutaneous implant placement with the benefits of the submuscular implant position traditionally used today.^6^ Applied as an extension of the pectoralis major muscle, ADM enlarges the subpectoral pocket, facilitating larger fixed-volume implants and potentially allowing 1-stage reconstructions.^7^ Early reports stated several benefits, including superior cosmetics, less need for tissue expanders, fewer elective reoperations, and less capsular contracture.^8,9^ However, ADM’s proposed advantages have not been universally accepted, and further cause for doubt was created by reports concerning harm, specifically higher rates of infection and implant loss.^10,11^ While biological meshes are approved by the Food and Drug Administration (FDA) in the US for reconstructive purposes, such as repair for abdominal hernias, ADM has not reached approval for use in breast reconstruction, and is regarded as off-label.^12,13,14^

IBBR typically requires multiple procedures with refinements and revisions for completion and to maintain aesthetics over time.^15,16,17^ The high rate of revision surgery becomes particularly evident in the setting of breast cancer treatment, where unilateral breast reconstructions frequently require contralateral procedures for symmetry. The increased use of postmastectomy radiotherapy also adds to the risk of revision surgery because of the risk of capsular contracture, leading to discomfort and poorer cosmetic outcomes.^16,18,19,20^

This open-label, multicenter, randomized clinical trial performed in Sweden and the United Kingdom aimed at evaluating ADM-assisted IBBR in the setting of breast cancer care. As a primary trial end point, we hypothesized that ADM would reduce the number of secondary surgeries compared with IBBR without ADM when measured at 24 months after the initial reconstruction. As secondary trial end points, we assessed patient-reported satisfaction and health-related quality of life (HRQoL). We have previously published 6-month early safety data on harm and HRQoL.^21,22^ Here we present reoperation rates and patient-reported outcomes at the primary trial end point of 24 months.

## Methods

### Study Design and Patients

The study was a prospective, multicenter, randomized clinical trial. Potential participants were identified by local investigators and enrolled from 5 different units in Sweden and United Kingdom. Women with confirmed invasive or preinvasive breast cancer planning for immediate IBBR with skin- or nipple-sparing mastectomy were eligible for inclusion. Exclusion criteria were previous radiotherapy to the breast region (anticipated need for adjuvant radiotherapy did not exclude patients), neo-adjuvant treatment with chemotherapy, smoking, a body mass index of 30 or above (calculated as weight in kilograms divided by height in meters squared), predicted implant size less than 200 mL or greater than 600 mL, pregnant or lactating women, insulin-dependent diabetes or any immunosuppressive disorder, allergy to porcine material or refusing to receive porcine material, or being unable or unwilling to provide written informed consent.

The study protocol was approved by the Central Ethical Review Board in Stockholm, and conducted according to the Declaration of Helsinki.^23^ A separate ethical approval was obtained for the study center in the UK (IRAS project ID: 150240). Written informed consent was obtained from all participants prior to any study-related procedures. This study followed the Consolidated Standards of Reporting Trials (CONSORT) reporting guideline.

### Randomization, Allocation, and Masking

Participants were randomized to either immediate IBBR with ADM (Acelity) and partial muscle coverage or to immediate IBBR without ADM using complete muscular coverage of the implant. Allocation to treatment was done using permuted block technique with random block size of 4 and 6. The randomization was computer based using a software module (Dynareg Systems) and stratified between centers to ensure balance between treatment arms. The trial inclusion and exclusion criteria were automatically verified by the computer program when randomizing a participant. Each participant was assigned a unique case number and recorded in a screening log kept locally. Physicians recruiting patients did not have access to screening log. The study was open label, with both surgeons and patients being informed about the allocation before their surgical procedure but concealed to participant until completion of baseline questionnaires to reduce allocation bias.

### Procedures

All patients underwent skin- or nipple-sparing mastectomy. The reconstruction was performed by a breast or plastic surgeon experienced with IBBR and familiar with the use of ADM. In the ADM group, the inferior insertion of the pectoralis major muscle (PMM) was detached from the chest wall after mastectomy, and the ADM sutured to its lower border and fixed along the inframammary fold, creating the implant pocket. For the control group, the PMM, serratus anterior muscle or fascia and, when needed, the rectus fascia was raised after mastectomy, allowing for an implant pocket with complete muscular and/or fascia coverage. The surgeon had the option of placing a definitive gel implant or using a tissue expander in both groups (technical details and trial protocol in Supplement 1 and eMethods in Supplement 2).

### Primary End Point

The primary trial end point was the number of surgical breast procedures at 24 months after the initial reconstruction (ie, reoperations). Secondary outcomes were health-related quality of life (HRQoL). Safety outcomes have previously been reported.^21^ Here, in addition to the primary outcome measure, HRQoL, we report patient-reported cosmetic outcomes. All outcomes were prespecified.

### Outcomes and Questionnaires

Reoperations were defined as procedures requiring general anesthesia in theater, such as capsulotomy with or without implant exchange, wound debridement with or without reentering of implant cavity, and implant removal (study protocol in Supplement 1).

The European Organization for Research and Treatment of Cancer Quality of life Questionnaire C30 (EORTC QLQC30) measures quality of life in cancer patients in clinical trials.^22,24^ The EORTC QLQ Breast cancer module QLQ-BR23 constitutes 5 multi-item scales assessing disease symptoms such as arm and breast symptoms, side effects of treatment, body image, and sexual functioning. Sexual enjoyment, hair loss, and future perspectives are measured by single items.^22,25^ The EORTC QLQ-BRR26 assesses satisfaction with results after breast reconstruction.^22,25^ Questionnaires were administered at baseline (prerandomization), and at 3 follow-up time points: 6, 12, and 24 months postreconstruction (eTable in Supplement 2).

### Statistical Analysis

Calculations of the study sample size were made with respect to the primary trial end point. A reoperation rate of 60% in the control group and 30% in the study group was estimated over the course of 24 months from the primary procedure. Our hypothesis was that the use of ADM in immediate IBBR would permit more single-stage procedures, resulting in fewer reoperations such as implant exchanges and revision surgery. Reoperation rate was deemed as a reasonable objective measure of surgical intervention required to maintain aesthetics and function in IBBR. To detect a statistical difference between the 2 treatment groups required in 98 total patients, the statistical significance level was set to 5% with a study power of 80%. To account for a loss to follow-up, 120 patients were intended to be recruited.

The difference of complication rates between the 2 groups are presented as differences in percentages together with 95% CIs. Differences were tested using a χ^2^ test or Fisher exact test when appropriate. Descriptive statistics such as means, standard deviations, and counts and percentages were used to describe patient demographics and outcomes.

Differences between treatment arms for HRQoL were estimated and tested using linear regression models, with subscales as dependent variables and allocation group as the independent variable. Results from these models are presented as mean differences (MD) together with 99% CIs. The level of significance was set to *P* < .01 as determined in Wald tests. Results from the EORTC questionnaires were analyzed according to the user instructions. Clinically relevant differences were determined as follows: 5 to 9 as small, 10 to 19 as moderate, and 20 or greater as large differences. All analyses used STATA version 15 (StataCorp) and SPSS version 25 (IBM Corp) for all analyses.

## Results

**Figure 1.**
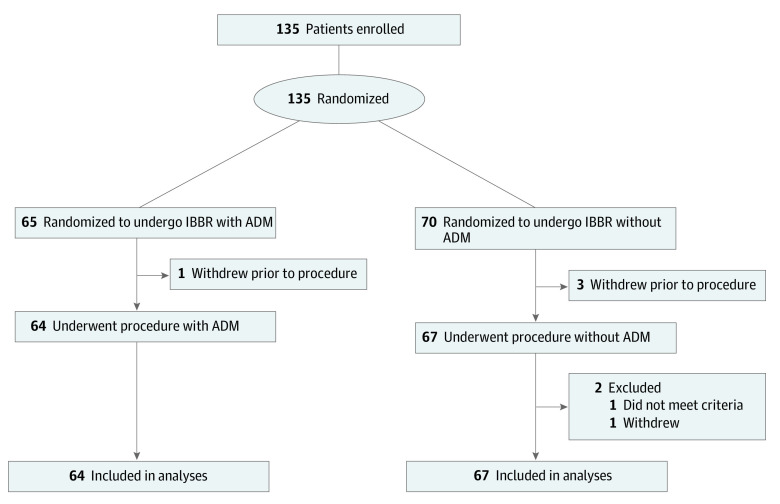
Trial Flowchart

Both groups exhibited similar baseline demographics (Table 1), with a mean (SD) age of 50.4 (9.5) years and a mean (SD) body mass index (BMI; calculated as weight in kilograms divided by height in meters squared) of 23.4 (2.7). From April 24, 2014, to May 10, 2017, 135 participants were consented and randomized to either immediate IBBR with ADM (65 participants) or IBBR without ADM (70 participants). At close of trial, 129 participants were available for analysis—64 in the study group and 65 in the control group (Figure 1).

**Table 1.** Baseline Characteristics

Characteristic	Patients, No. (%)	
	IBBR with ADM (n = 64)	IBBR without ADM (n = 65)
Patient demographic data		
Age, mean (SD), y	51.8 (9.5)	49.1 (9.4)
BMI, mean (SD)	23.6 (2.6)	23.0 (2.7)
Invasive ductal cancer	32 (50)	28 (43)
Invasive lobular cancer	13 (20)	14 (22)
DCIS	17 (27)	23 (35)
Paget’s disease of the breast	2 (3)	0
Treatment related variables		
Axillary surgery	64 (100)	63 (97)
Sentinel node only	52 (81)	57 (88)
Axillary lymph node clearance^a^	12 (19)	6 (9)
Nipple sparing mastectomy	26 (40)	32 (51)
Mastectomy weight, mean (SD), g	358.4 (161.5)	342.4 (156.9)
Adjuvant radiotherapy	29 (45)	35 (54)
Adjuvant chemotherapy	33 (52)	30 (46)
Allocation-related variables^b^		
Direct-to-implant^c^	39 (61)	11 (17)
Implant volume, mean (SD), mL	313.6 (66.6)	255.9 (46.9)
Expander volume, mean (SD), mL	445.2 (94.4)	383.6 (83.2)
Intraoperative filling volume, mean (SD), mL	149.8 (64.3)	112.1 (51.8)

Abbreviations: ADM, acellular dermal matrix; BMI, body mass index (calculated as weight in kilograms divided by height in meters squared); DCIS, ductal carcinoma in situ; IBBR, implant-based breast reconstruction.

^a^ With or without previous sentinel node.

^b^ Variables dependent on allocation group. All allocation-related variables *P* < .001 in *t* tests for continuous variables and Fisher exact test for categorical variables.

^c^ Fixed-volume implant at time of mastectomy.

Both groups exhibited similar baseline demographics with adjuvant therapy equally distributed between the study and control group (mean [SD] age: ADM group, 51.8 [9.5] years vs without ADM, 49.1 [9.4] years; BMI: ADM, 23.6 [2.6] vs without ADM, 23.0 [2.7]) (Table 1). In the ADM group, 31 patients (48%) had at least 1 reoperation on the ipsilateral side vs 35 (54%) in the control group (*P* = .54) (Table 2). The corresponding number of reoperations for the contralateral side was 34 (53%) vs 31 (48%), respectively (*P* = .54). The total number of anesthetic events also exhibited equal distribution between the groups, with 44 patients (69%) having at least 1 anesthetic event in the ADM group compared with 43 (66%) in the control group (*P* = .75) (Table 2). Two patients in the ADM group and 3 patients in the control group had a risk-reducing mastectomy on the contralateral side, with all 5 surgeries included in the final analysis.

**Table 2.** Number of Reoperations and Procedures per Patient and Study Arm Performed Under General Anesthesia on the Ipsilateral and Contralateral Breast at 24 Months

**Characteristics**	**Patients, No. (%)**		**Difference in percentages (95% CI)**	***P* value**
	**IBBR with ADM (n = 64)**	**IBBR without ADM (n = 65)**		
**Ipsilateral side**				
No. of reoperations				
1	21 (33)	28 (43)	NA	NA
2	9 (14)	6 (9)	NA	NA
3	1 (2)	1 (2)	NA	NA
Subtotal procedures per study arm (primary trial end point)	42	43	NA	NA
No procedure on ipsilateral side	33 (52)	30 (46)	NA	NA
Any procedure on ipsilateral side	31 (48)	35 (54)	−5.4 (−22.6 to 11.8)	.54
**Contralateral side**				
No. of reoperations				
1	31 (48)	25 (38)	NA	NA
2	2 (3)	5 (8)	NA	NA
3	1 (2)	1 (2)	NA	NA
Subtotal procedures per study arm (primary trial end point)	38	38	NA	NA
No procedure on contralateral side	30 (47)	34 (52)	NA	NA
Any procedure on contralateral side	34 (53)	31 (48)	5.4 (−11.7 to 22.7)	.54
**Total procedures**				
Total ipsilateral and contralateral procedures per study arm (primary trial end point)	80	81	NA	NA
Anesthetic events per patient				
1	30 (47)	29 (45)	NA	NA
2	11 (17)	11 (17)	NA	NA
3	3 (5)	3 (5)	NA	NA
Total anesthetic events per study arm	61	60	NA	NA
No event	20 (31)	22 (34)	NA	NA
Any event	44 (69)	43 (66)	2.6 (−13.6 to 18.8)	.75

Abbreviations: ADM, acellular dermal matrix; IBBR, implant-based breast reconstruction; NA, not applicable.

Nine patients (14%) in the ADM group had the implant removed. Four losses came within 6 months after the initial IBBR with ADM following early surgical complications. Another 4 implant removals followed after exchange with pocket revision, 2 after a deep wound infection, and 2 due to persistent seroma with chronic skin redness but without infection signs (all 4 had received adjuvant radiotherapy). One further patient had the implant removed following a local cancer recurrence (Table 3).

**Table 3.** Type and Number of Reoperations Performed Under General Anesthesia

Characteristics	Procedures, No. (%)	
	IBBR with ADM (n = 80)	IBBR without ADM (n = 84)
Ipsilateral side		
Implant exchange including capsulotomy	20 (25)	24 (29)
Revision with implant exchange^a^	4 (5)	0
Abdominal advancement and implant exchange	2 (3)	2 (2)
Capsulotomy (without implant exchange)	2 (3)	8 (10)
Removal of ADM	1 (1)	0
Capsulotomy with fat transplantation	1 (1)	0
Implant removal	9 (11)	7 (8)
Re-excision after positive margins	0	1 (1)
Evacuation of hematoma	3 (4)	1 (1)
Secondary reconstructions after implant removal		
Expander implant	0	1 (1)
Autologous DIEP	0	1 (1)
Combined autologous/implant	0	1 (1)
Contralateral side		
Mammoplasty^b^	19 (24)	23 (27)
Augmentation with or without mammoplasty	15 (19)	11 (13)
Implant exchange including capsulotomy	1 (1)	1 (1)
Risk-reducing mastectomy and IBBR	2 (3)	3 (4)
Implant removal^c^	1 (1)	0

Abbreviations: ADM, acellular dermal matrix; DIEP, deep inferior epigastric perforators; IBBR, implant-based breast reconstruction.

^a^ Procedure due to an adverse surgical event.

^b^ Reduction of mammoplasty or mastopexy.

^c^ Augmentation implant removed because of patient preference.

In the control group, 7 patients (11%) had the implant removed. Four reconstructive failures were due to early surgical complications, all within 6 months from the initial IBBR without ADM. The remaining 3 had the implant removed because of patient preferences, and later converted to a delayed reconstruction with a 2-stage expander-implant or autologous tissue (Table 3).

**Figure 2.**
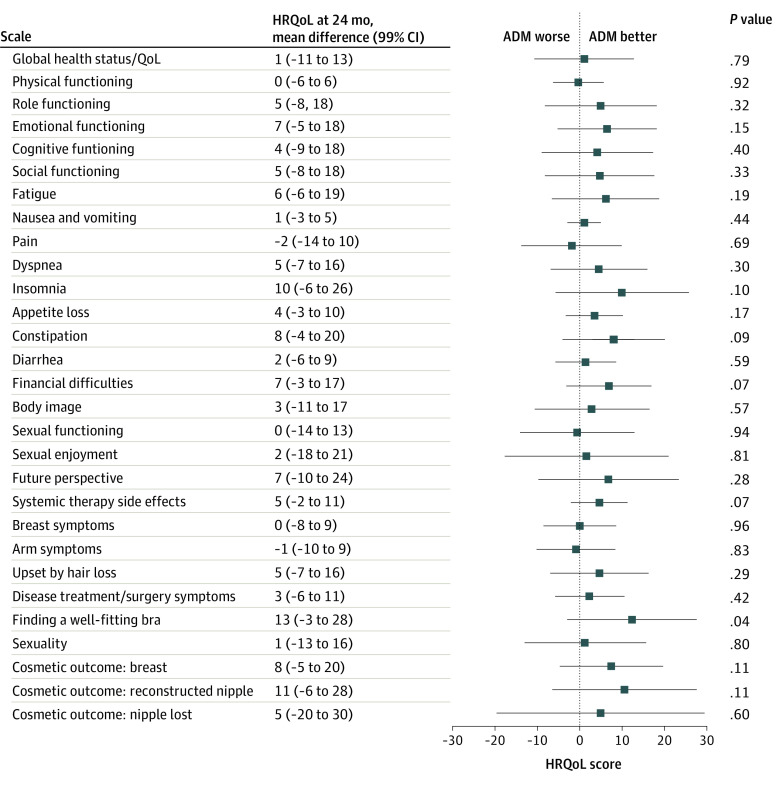
Health-Related Quality of Life (HRQoL) Difference Between Implant-Based Breast Reconstruction With and Without Use of an Acellular Dermal Matrix

At 24 months, no statistically significant differences between the groups were detected concerning HRQoL, measured with QLQ-C30, QLQ-BR23, and QLQ-BRR26 (Figure 2). For the breast reconstruction–specific questionnaire (QLQ-BRR26) at 24 months assessment, the cosmetic outcome subscale yielded a mean (SD) score of 68 (23) for the ADM group and 60 (24) for the control group, with a mean difference of 8 (99% CI, −5 to 20, *P* = .11), corresponding to a small clinically meaningful difference (Figure 2).^26^ For problems finding a well-fitting bra, scores were also in favor of the ADM-group compared with the control group, with a mean (SD) score of 19 (25) and 31 (32), respectively, and a mean difference of −13 (99% CI, −28 to −3; *P* = .04), a moderate clinically meaningful difference (Table 2).^26^ For all other domains, no statistically significant or clinically meaningful differences were found (eTable in Supplement 2).

## Discussion

Implant-based breast reconstructions commonly involve additional surgeries to reach completeness.^27^ We performed this randomized trial to clarify the role of ADM, postulating that ADM-assisted IBBR would reduce the number of reoperations compared with conventional IBBR within a follow-up time of 24 months, including less need for elective revisional surgery. As a secondary aim, we assessed whether ADM yielded superior HRQoL and patient-reported cosmetic outcomes.

Immediate IBBR with ADM did not generate fewer reoperations, superior HRQoL results, or patient-reported cosmetic outcomes over 24 months of follow-up compared with conventional IBBR without ADM. We were therefore unable to confirm proposed advantages of ADM-augmented IBBR.

With the introduction of biological meshes for IBBR in 2005, the shortcomings of using implants in breast reconstructions were hoped to be alleviated.^28^ With improved cosmetic outcomes, more single-stage surgeries, and possibly fewer reoperations, it was hoped ADM would reduce the surgical burden for women undergoing IBBR. Despite widespread use and promising early data, a lack of clear evidence validating the potential advantages of ADM use remains unresolved.^9,29^

Outcomes from the Mastectomy Reconstruction Outcomes Consortium Study (MROCS),^30^ a large prospective cohort study recruiting nearly 2000 patients, revealed that 46% of patients undergoing implant-based reconstructions had a secondary surgical procedure within a 2-year follow-up time. For the 1-stage IBBR cohort, the reoperation rate was 33%, including revisions following unanticipated adverse events. The average number of secondary surgeries in that study ranged from 1.4 procedures in single-stage procedures to 2.4 in tissue-expander and implant reconstructions. The MROCS included both implant- and autologous-based modalities. Similar revision rates were reported from a retrospective single-institution study, revealing a 5-year revision rate of 21% for direct-to-implant reconstructions with ADM, and 20% for tissue and expander procedures.^31^ A further single-institution study on expander and implant procedures reported a revision rate of 38% for expander and implant procedures at 2 years.^32^ A retrospective cohort study with 4000 patients found that 88% underwent at least 1 reoperation over 5 years, including both autologous and prosthetic modalities as well as immediate and delayed procedures.^15^ In summary, secondary procedures following breast reconstructions are common, and women should be advised to expect on average over 2 reoperations over a 5-year period, with rates increasing in the presence of surgical complications and postoperative radiotherapy.

With several ipsilateral and contralateral reoperations done concomitantly, we also show the total number of anesthetic events. Thirty-nine patients in the ADM group were reconstructed with a fixed-volume implant, with 15 of these having at least 1 additional reoperation. Corresponding numbers for the non-ADM group were 11 and 5 respectively. As 1-stage reconstructions offer the opportunity to recreate the breast in a single procedure, this would potentially translate to fewer operations. However, the disadvantage of a direct-to-implant procedure is that it does not entail an opportunity for secondary adjustments, while tissue expanders as 2-stage surgeries permit alterations for symmetry during the second surgery. The majority of secondary procedures in this trial were done for the purpose of symmetry, commonly with a contralateral mammoplasty, as well as releasing a capsular contracture on the ipsilateral side in conjunction with an implant exchange. It has been suggested that 1-stage procedures pose potentially greater risks for complications and reoperations in ADM-assisted IBBR.^10^ A 2021 randomized clinical trial^33^ comparing biological and synthetical meshes in IBBR showed more complications for ADM, including loss of implant, compared with synthetic matrices. Outcomes from the MROCS study and a randomized trial from the Netherlands also align with our previously published results on adverse events, in which ADM was associated with significantly more surgical complications, including reconstructive failure with loss of implant.^10,12,21^

In addition to evaluating the rates of revision surgery, we assessed if ADM could improve patient-reported cosmetic outcomes in IBBR. If the aim of the procedure is to restore form and function, measuring HRQoL and aesthetic outcomes after reconstructive procedures is central. Several retrospective studies have reported improved aesthetic outcomes with ADM-assisted IBBR.^34,35^ However, most results are based on assessments by the surgeons, which carries a risk of bias, so that suggested benefits may not necessarily agree with the patient’s perspective. Our results indicated a clinically relevant minor advantage for a couple of domains for ADM, while the remainder showed no statistically or clinically significant difference between the treatments.

HRQoL and aesthetic outcomes from our trial are comparable with previously published data from the Breast Reconstruction in One Stage study (BRIOS), a randomized trial evaluating fixed-volume implants with ADM, including therapeutic and risk-reducing surgeries. Findings from the BRIOS study could not confirm HRQoL advantages nor differences in patient-reported satisfaction with cosmetic results or aesthetic results judged from photos by a panel of surgeons.^29^ Similarly, the MROC study did not identify any significant improvements in patient-reported outcomes when using ADM.^12^

### Limitations

While our study, performed in a randomized setting, could not verify benefits of ADM-assisted IBBR compared with traditional muscle coverage, it is important to point out several limitations in this trial. First, to avoid interference with postmastectomy radiotherapy, implant-expanders with peripheral injection ports are commonly used at our institutions. These tissue expanders are also designed to work as permanent implants. However, contralateral procedures present an opportunity to concurrently revise the ipsilateral reconstruction, frequently resulting in an exchange to a fixed-volume prosthesis or modification of implant size after a direct-to-implant procedure. This essentially eliminated the benefit of ADM in reducing reoperations in this study. In a setting of risk-reducing surgery with bilateral mastectomies, the advantage of single-stage reconstructions might be more noticeable. Second, adjuvant radiotherapy following IBBR is a known predictor for surgical complications and revision surgery.^36^ Forty-five per cent of the patients in the ADM group had postoperative radiotherapy vs 54% in the non-ADM group. While ADM has been suggested by several authors to mitigate capsular contracture, our study was not designed, nor had the power, to evaluate the specific role of ADM in the setting of adjuvant radiotherapy.^37^ Third, this current trial was initiated before the shift to the subcutaneous implant position, known today as the prepectoral method, became repopularized. The modern prepectoral technique usually involves a biological or synthetic mesh to control the implant pocket, as well as allowing for 1-stage procedures with larger fixed-volume implants.^38^ In light of this, the partial- and full-muscle coverage techniques used in this trial could be viewed as less contemporary surgical methods in today’s breast reconstruction setting. It is worth mentioning that there is a lack of robust data to evaluate the prepectoral technique, especially in the setting of breast cancer treatment.

We elected to present aesthetic outcomes based on the point of view of patients rather than from external observers. However, without a direct reference, patients might be similarly pleased with the overall experience irrespective of the surgical method. Physicians evaluating postoperative photos might therefore appreciate improved aesthetic results differently from patients based on their collective references. Nonetheless, cosmetics are difficult to measure, and outcomes judged by the patient might not align with views by the profession.

While the randomization result was concealed to patients until after completion of baseline questionnaires, allocation could in some part have influenced how women scored their satisfaction with the reconstructive outcome. Similarly, although most surgeons are aware that mastectomy flap viability can affect the outcome of ADM-assisted IBBR, the setting of cancer treatment commonly requires a “therapeutic mastectomy approach,” which would somewhat limit the surgical control of the flap thickness compared with a risk-reducing procedure.

Finally, with expectations initially set high, our hypothesis that ADM would noticeably reduce reoperation rates might have been optimistic. However, this assumption was also balanced against what needed to be pragmatic considerations about sample size, study time, and costs. While there were data available on reoperation rates for IBBR with full-muscle coverage, there were no available data on the possible impact of ADM on reoperation rates when this study was designed. Since then, reports on revision surgery following IBBR with ADM have been published.^12^

## Conclusions

Our present and previous results published from this trial do not support an advantage of using an acellular dermal matrix in IBBR. Immediate IBBR with ADM did not yield fewer reoperations compared with conventional IBBR without ADM, nor was IBBR with ADM superior in terms of HRQoL or patient-reported cosmetic outcomes. Patients treated for breast cancer contemplating ADM-supported IBBR should be informed about the lack of evidence validating ADM’s suggested benefits.

## Notes

Supplement 1.
Trial Protocol

Supplement 2.
eMethods.
**eTable.** Patient Reported Scores at 24 Months for the Breast Reconstruction-Specific EORTC-BRR26 Questionnaire

Supplement 3.
Data Sharing Statement
