# Supplementary material for: Effect of Immediate Implant-Based Breast Reconstruction After Mastectomy With and Without Acellular Dermal Matrix Among Women With Breast Cancer: A Randomized Clinical Trial
Source: JAMA Netw Open. 2021 Oct 1;4(10):e2127806. doi: 10.1001/jamanetworkopen.2021.27806 (PMC8486981; doi:10.1001/jamanetworkopen.2021.27806)
Supplement: Supplement 1. — Trial Protocol [file jamanetwopen-e2127806-s001.pdf]

Lohmander F, Lagergren J, Johansson H, Roy PG, Brandberg Y, Frisell J. Effect of immediate implant-based breast reconstruction after mastectomy with and without acellular dermal matrix among women with breast cancer. *JAMA Netw Open.* 2021;4(10):e2127806. doi:10.1001/jamanetworkopen.2021.27806

Immediate Breast Reconstruction with or without the use of Acellular Dermal Matrix (Strattice™) - A Randomized Controlled Trial

Sponsor: Karolinska University Hospital, Sweden
Funder: LifeCell™ EMEA Limited
Protocol: Version 1.0 – 30th April 2013

Collaborators: Karolinska University Hospital, Sweden
Karolinska Institute, Sweden
LifeCell™ EMEA Limited

REC No.: TBC
ClinicalTrials.gov: NCT02061527

This protocol was designed and will be conducted, recorded and reported in compliance with the principles of Good Clinical Practice (GCP) guidelines.

CONTACTS PAGE

Chief Investigator, Oxford:

Pankaj G. Roy, MD, PhD.
Consultant Oncoplastic Breast Surgeon.
Oxford University Hospitals NHS Foundation Trust
Oxford, United Kingdom
Phone: +44 (0)1865 220935
Email: pankaj.roy@ouh.nhs.uk

Lead Investigators, Sweden:

Fredrik Lohmander, MD
Department of Breast and Endocrine Surgery Karolinska University Hospital and Karolinska Institute Stockholm, Sweden
Tel No: +46 709370721
Email: fredrik.lohmander@ki.se

Jakob Lagergren, MD, PhD.
Department of Plastic and Reconstructive Surgery
Karolinska University Hospital and Karolinska Institute
Stockholm, Sweden
Tel No: +46 8 51779659
Email: jakob.lagergren@karolinska.se

Catharina Eriksen, MD, PhD.
Department of Surgery
Capio St: Görans Hospital and Södersjukhuset AB
Stockholm, Sweden
Tel No: +46-70-481-07-32
Email: catharina.eriksen@capio.se

Jan Frisell, MD, PhD,

Institute for Molecular Medicine and Surgery
Karolinska University Hospital
Stockholm, Sweden.
Tel No: +46-8-51770915
Email: jan.frisell@ki.se

STUDY SYNOPSIS

**Study Title:**	Immediate Breast Reconstruction with and without the use of Acellular Dermal Matrix - A Randomized Controlled Trial
**Short title:**	ADM Strattice™ Breast Trial
**Study design:**	A randomized controlled multicentred trial, comparing Immediate Breast Reconstruction (IBR) without (group A) and with (group B) the use of Acellular Dermal Matrix (Strattice™), in the setting of breast cancer treatment.
**Study population:**	Patients with breast cancer who are having a planned mastectomy and Immediate Breast Reconstruction with implants. The study will aim to recruit 120 patients from 5 centres across the UK and Sweden.
**Primary objective:**	The purpose of this study is to evaluate the effectiveness of Acellular Dermal Matrix (Strattice™) in post-mastectomy Immediate Breast Reconstruction (IBR) with implants, in the setting of breast cancer treatment.
**Primary outcome:**	The primary outcome measure is a comparison of the number of unplanned surgical breast procedures for each patient between the two treatment arms.
**Secondary outcomes:**	• Aesthetic outcome<br>• No. of surgical procedures<br>• Complications<br>• Cost-Benefit analysis

	• Quality of Life
**Study duration:**	24 months total study duration. Study duration for each patient will be 24 months from time of initial surgery.

TRIAL SCHEMA

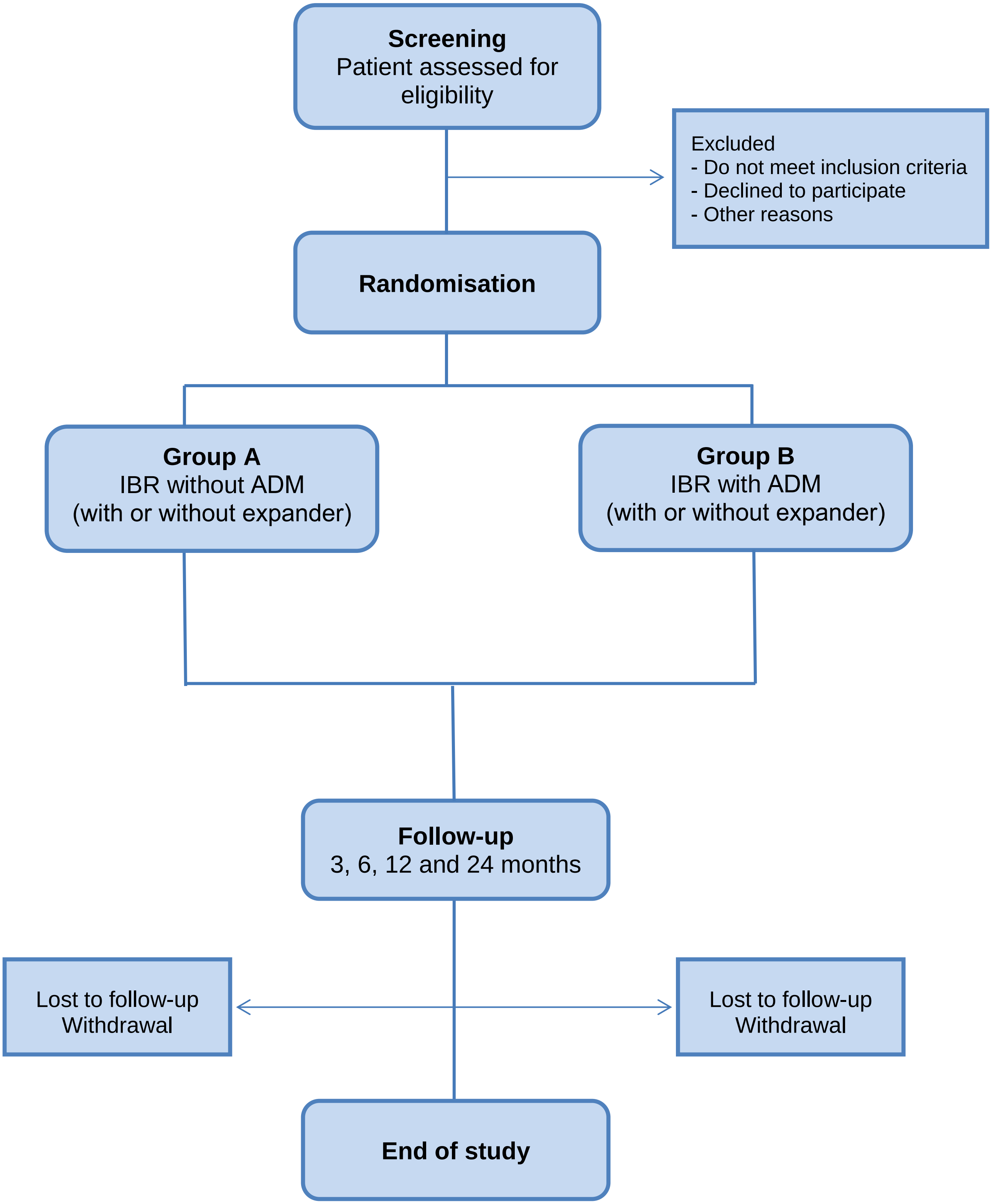

SCHEDULE OF EVENTS

	Screening	Visit 0 Baseline	Surgery / Post-op[2]	Visit 1 (1-4 weeks)	Visit 2 (3 months)	Visit 3 (6 months)	Visit 4 (12 months)	Visit 5 (24 months)
PIS provided	x							
ICF signed	x							
Patient randomised	x							
Eligibility checked	x	x						
Medical history		x						
Clinical examination / measurements		x	x	x	x	x	x	x
Drain removed				x				
Photographs taken		x			x	x	x	x
Case Report Form (CRF)		x	x	x	x	x	x	x
QoL Questionnaire - EORTC QLQ-C30		x[1]				x	x	x
QoL Questionnaire – EORTC BR23		x[1]				x	x	x
QoL questionnaire – EORTC BRR26						x	x	X
Adverse Events			x	x	x	x	x	X

[1] – Baseline QoL questionnaires should be completed before randomization
[2] – Patients undergoing single-Stage direct to implant IBR will have one surgical visit. Patients undergoing two-stage IBR with expander will attend two separate surgical visits.

ABBREVIATIONS

Abbreviation	Explanation
ADM	Acellular Dermal Matrix
AE	Adverse Event
BCS	Breast Conserving Surgery
CC	Capsular Contracture
CRF	Case Report Form
EORTC	European Organisation for Research and Treatment of Cancer
GCP	Good Clinical Practice
IBR	Immediate Breast Reconstruction
ICF	Informed Consent Form
IP	Intellectual Property
ISF	Investigator’s Site File
MDT	Multi-disciplinary Team Meetings
NHS	National Health Service
NRES	National Research Ethics Service
OUH	Oxford University Hospitals
PIS	Patient Information Sheet
PMM	Pectoralis Major Muscle
QoL	Quality of Life
R&D	Research and Development
REC	Research Ethics Committee
RT	Radiotherapy
SAE	Serious Adverse Event
SMF	Sub-mammary fold
SSDL	Site Signature and Delegation Log
TE	Tissue Expanders
TRAM	Transverse Rectus Abdominis Musculocutaneous
UK	United Kingdom

CONTENTS

SIGNATURE PAGE .... **Error! Bookmark not defined.**
CONTACTS PAGE ii
STUDY SYNOPSIS iii
TRIAL SCHEMA v
SCHEDULE OF EVENTS vi
ABBREVIATIONS vii
CONTENTS viii
3 BACKGROUND AND TRIAL RATIONALE 1
4 STUDY SETTINGS 4
5 TRIAL DESIGN AND METHODOLOGY 5
- 5.1 Study population 5
- 5.2 Study duration 5
- 5.3 Randomisation 5
- 5.4 Statistical Methods 5

6 OUTCOMES AND ASSESSMENT OF EFFICACY 7
- 6.1 Primary outcome measures 7
- 6.2 Secondary outcome measures 7

7 ELIGIBILITY CRITERIA FOR PARTICIPANTS 8
- 5.1 Inclusion Criteria 8
- 5.2 Exclusion Criteria 8

8 STUDY VISITS / PROCEDURES 9
- 6.1 Screening 9
- 6.2 Informed consent 9
- 6.3 Randomisation 10
- 6.4 Study settings and procedures 11
- 6.5 Schedule of Events 12
- 6.6 Study completion 13
- 6.7 Study withdrawal 13

9 Study Data Collection 15
- **9.1 Case Report Forms (CRF's)** 15
- 9.2 Quality of Life Questionnaires 15
- 9.3 Photographs 15

10 STUDY MANAGEMENT 17
- 10.1 Data recording 17

10.2 Protocol deviations....18
10.3 Monitoring and auditing....18
10.4 Archiving....18
11 Safety Reporting....19
11.1 Adverse Events....19
11.2 Serious Adverse Events....19
9.3 Reporting procedure....20
10. ETHICS....21
11. Data and Publication policy....22
11.1 Data and Intellectual Property....22
11.2 Confidentiality and Data Protection....22
12 Insurance and indemnity....24
13 Funding....24
14 REFERENCES....25
15 APPENDICES....27
15.1 Appendix (1): Definition of reoperations....27
15.2 Appendix (2): Instructions for Randomisation....28
15.3 APPENDIX (3): Surgical technique using ADM (Strattice™) in IBR [11]....29
15.4 APPENDIX (4): Standard procedures for taking photographs....31
15.5 APPENDIX (5): Defining a surgical infection....32

3 BACKGROUND AND TRIAL RATIONALE

Despite the availability of Breast Conserving Surgery (BCS), removal of the breast is performed in 40-50% of breast cancer patients. There has been a growing demand for breast reconstruction, particularly as an Immediate Breast Reconstruction (IBR) at the time of the mastectomy rather than a delayed procedure after mastectomy. IBR after mastectomy has its advantages over delayed, as it is possible to keep more of the native skin envelope intact, and as such, creating a more natural shape. It also carries psychological benefits for women [1].

In broad terms, there are three different techniques in breast reconstruction available: (1) Tissue expander/implant reconstruction. (2) Pedicled flaps with or without an implant. (3) Transfer of abdominal fat in the form of a transverse rectus abdominis musculocutaneous or TRAM flap, or (today more commonly) as a free perforator flap using the same abdominal skin and fat but no muscle, based on deep inferior epigastric perforator vessels (DIEP). Other alternatives, but less common, are free flaps using buttock or thigh fat (SGAP Flap). Tissue expander/ implant reconstruction remains the most common method, especially in the setting of IBR [2 3].

Two variations of the technique for IBR with implants can be applied: The partial muscle coverage (dual plane) and the full submuscular technique. With partial muscle coverage the implant is placed under the pectoralis major muscle (PMM), which leaves the inferior and lateral part of the implant with skin and subcutaneous tissue coverage only. With full muscle coverage, the implant is completely covered by the PMM, anterior rectus fascia and usually a serratus anterior muscle flap. Full submuscular coverage has traditionally been recommended to prevent direct contact between the implant and the mastectomy skin flaps, but can result in an implant positioned too high and at times a more difficult lower pole expansion with a less defined submammary fold (SMF), compared with placing the implant in a partial subcutaneous position (dual-plane) [4]. It also requires a more extensive muscle dissection, with longer operating time. The partial muscle technique also has its limitations with migration of implant, bottoming out, malposition, as well as implant visibility and palpability [5].

Prosthetic breast reconstruction has drawbacks, where capsular contracture (CC) remains the most common problem. CC can severely impair the aesthetics of the reconstruction and give patients discomfort. CC is especially problematic in patients receiving adjuvant radiotherapy (RT), and with few exceptions, these patients need further surgical revisions, and on occasion conversion to autologous flap procedures. Currently about 40% of the patients receive postmastectomy RT at our institutions.

The incidence of CC reported in the literature ranges from 15%-100% in the presence of adjuvant RT. The occurrence of CC is unpredictable and revision surgery such as capsulotomy or capsulectomy may not prevent its recurrence. In the long term, this creates a high incidence of reoperations for implant-based reconstructions, especially in the presence of RT, to maintain an acceptable aesthetic appearance and also comfort of the reconstructed breast. The incidence of revision surgery reported in the literature greatly varies, where some studies report a reoperation rate as high as 70%. Over the years, with the introduction of different skin-sparing and nipple-sparing mastectomy techniques, coupled with better implant devices, there has been an improvement in the aesthetic results of prosthetic breast reconstruction. However, the rate of reoperation remains high.

To utilize the benefits of the dual-plane technique, perform more reconstructions as a single-stage procedure without tissue expansion, and overcome the drawbacks of using full muscle coverage, Acellular Dermal Matrix (ADM) in implant-based breast reconstruction was introduced in 2003. ADM is a biological product with different origins, depending on the manufacturer. This study will be using Strattice™, an ADM product marketed by Lifecell™ EMEA Limited derived from porcine dermis. The dermal tissue undergoes several processes to reduce antigens, remove cells and DNA, while maintaining an intact extracellular matrix (ADM). The remaining matrix allows for vascular ingrowth and integration into host tissue [6].

The use of ADM has quickly gained acceptance, with the following benefits reported in the literature: decreasing or eliminating the need for tissue expanders (TE), improved aesthetic outcome, increased initial fill volumes of TE, fewer expansions, decreased rate of revision surgery and reoperations, decreased incidence of CC, better control of SMF, faster time to completion of reconstruction, improved lower pole expansion, and reduced costs.

There is supporting evidence for improved aesthetic outcome [7, 8, 9, 10] from several case series without comparison groups, with personal opinions and no data, and from single surgeons. Supporting evidence for decreasing or eliminating the need for tissue-expanders (TE) also comes from several review articles.[11, 12, 8, 13, 14, 15, 16, 17] For decreased rates of revision surgery there is some supporting evidence, as well as for decreased incidence of capsular contracture.[1, 7, 12, 15, 18]

As for improved lower pole expansion, there is no data. There are some articles supporting the evidence of reduced costs for an implant reconstruction [19].

One concern for using ADM in IBR is the possibility of complications. There is some conflicting data on the actual complications rates, and recent reports suggests a higher incidence of flap necrosis, seroma and infection.[20,21, 22, 23] Other groups report a complication rate similar to IBR without ADM.[24] A recent review and meta-analysis paper by Ho et al also suggests a significantly higher rate of complications, such as flap necrosis.[25] However, there is currently no data from any prospective randomized trials that supports the above described advantages or disadvantages.

4 STUDY SETTINGS

Currently in Oxford, most patients receiving Immediate Breast Reconstruction (IBR) using implants will undergo a two stage post-mastectomy reconstruction involving an initial procedure to fit a tissue expander and then a second surgery to replace the tissue expander with a permanent breast implant. This study will compare this standard treatment option with the additional use of ADM (Strattice™) as surgical mesh reinforcement.

Strattice^{TM} Tissue Matrix is a porcine dermis-derived biologic (pig skin) surgical mesh used in surgery to help support the lower outer part of the breast, providing a scaffold, or framework, which allows the body's own cells and blood vessels to grow into it. It gradually becomes part of the body as over time Strattice™ is replaced by the body's own tissue. Strattice^{TM} Tissue Matrix itself contains no human cells. Strattice^{TM} has been indicated for use in post-mastectomy breast reconstruction with implant since 2008 and carries a CE mark, which means it has been approved for use in the European Economic Area (EEA). It is not an experimental product. Strattice^{TM} is marketed by LifeCell^{TM} EMEA Ltd. who are providing the product free of charge for this study.

Strattice^{TM} is routinely used in breast reconstruction in some European countries, including in the UK, but is not routinely available for patients undergoing treatment for breast cancer in Oxford University Hospitals (OUH) NHS Trust due to cost implications. The results of this study may support a case for the routine use of the product if the cost of the product is offset by the reduction in number of subsequent operations or reduced hospital visits. The study will evaluate the two treatment arms in terms of effectiveness, safety and efficacy endpoints as well as economic considerations.

5 TRIAL DESIGN AND METHODOLOGY

The study is a randomized controlled multicentred trial comparing Immediate Breast Reconstruction (IBR) without (Group A) and with (Group B) the use of ADM (Strattice™). Participants eligible for the study are women with breast cancer who are having a planned mastectomy and IBR with implants.

5.1 Study population

The study will aim to recruit 120 patients from 5 different sites across the UK and Sweden: Karolinska University Hospital (Sweden), Capio St: Görans Hospital (Sweden), Danderyds Hospital (Sweden), Södersjukhuset (Sweden), Churchill Hospital (UK)

5.2 Study duration

The total study duration for each patient will be 24 months post initial surgery. The definition of end of study will be the last follow-up visit for the last patient recruited.

5.3 Randomisation

Patients will be allocated to treatment according to permuted block technique. The randomization will be performed using a computer based system located at Regional Cancer Center (RCC) within Karolinska Institutet. The randomization process will be stratified for the participating units, and also for patients treated with neo-adjuvant chemotherapy.

5.4 Statistical Methods

The main endpoint to be analyzed is the proportion of unplanned reoperations after the initial breast reconstruction. The difference in proportions between the two groups will be presented as a difference in proportion together with a 95% confidence interval. Differences will be tested using Fishers Exact Test. Logistic regression will be used to take into account possible confounding factor.

The sample size calculation is made with respect to the primary endpoint, which is to compare the number of unplanned surgical procedures (unanticipated) between IBR using full muscle coverage without ADM vs. IBR with ADM using partial muscle coverage (group B) over the study period of two years. The power calculation is based on a reoperation rate of 60% in the non-ADM group and a reoperation rate of 30% in the ADM group. The follow-up time is 24 months from the primary operation.

To detect a statistical difference between the two groups a total number of 98 patients - i.e. 50 patients in each arm – is needed, if the significance level is set to 5% and the power in the study to 80%. To account for a loss to follow-up, a total of 120 patients will be recruited in the study.

6 OUTCOMES AND ASSESSMENT OF EFFICACY

6.1 Primary outcome measures

The primary endpoint of this study is the number of unplanned/ unanticipated surgical breast procedures. Other endpoints in the trial will be secondary.

As reoperation rates are a relatively objective measure of surgical intervention required to attain and maintain acceptable aesthetics in implant based reconstructions, it will serve as a good primary endpoint for the study. See Appendix 1 for guidance on defining reoperations.

6.2 Secondary outcome measures

1. To measure any difference between the two study groups in complication rates.

2. To compare the total number of surgical procedures (planned or unplanned) between the two study groups).

3. To measure the aesthetic outcome between the two study groups using a 6-point scale. The evaluation will be done by three independent groups consisting of lay people, professionals (breast/ plastic surgeons) and patient evaluation. The aesthetic evaluation will be based on the pre and postoperative photos.

4. A cost-benefit analysis comparing the cost for an IBR with implant without ADM, vs. IBR with ADM (Strattice™) during a 24 months follow-up time.

5. Measure Quality of Life in both groups, using the EORTC QLQ-C30 (Generic tool), EORTC-BR23 (Breast cancer specific tool), EORTC-BRR26 (Specific for breast reconstruction)

7 ELIGIBILITY CRITERIA FOR PARTICIPANTS

5.1 Inclusion Criteria

1. Patient with invasive or pre-invasive (in situ) breast cancer, planned for immediate breast reconstruction post-skin sparing or nipple sparing mastectomy (unilateral or bilateral)
2. Patient agrees to participate in study and to sign an informed consent form
3. Able and willing to return for all scheduled and required study visits

5.2 Exclusion Criteria

1. Is a smoker (patient who has quit at least 2 weeks prior to surgery can be included)
2. BMI <18 or > 30
3. Previous radiation therapy to the area at any time (adjuvant or planned therapy is not an exclusion criteria)
4. Insulin-dependent diabetes or any immune deficiency requiring immunosuppressant use such as cortisone or biological therapies
5. Predicted **implant** size <200 or >600 ml per investigator assessment
6. Allergy to porcine or refuses to receive porcine material
7. Pregnancy or lactating
8. Neoadjuvant treatment with chemotherapy (adjuvant or planned therapy is not an exclusion criteria)
9. Patient is unable or unwilling to give informed consent.

8 STUDY VISITS / PROCEDURES

6.1 Screening

Potential participants will be identified by local investigators or suitably qualified members of the patient's clinical care team. This may be at routine clinics, during consultations for discussion of treatment or at multi-disciplinary team meetings (MDT's). The Patient Screening Log should be completed for all patients considered for the trial, indicating whether the patient was recruited to the study and if not the reason for this i.e. whether patient declined or was deemed ineligible.

Eligible patients will then be approached about the study at their routine clinic appointment and provided with a Patient Information Sheet (PIS). The local investigator or delegated individual will explain the nature, purpose, and possible benefits and risks of the trial to the patient. If the patient decides to take part in the study they will be asked to complete and sign an Informed Consent Form (ICF).

6.2 Informed consent

Informed consent must be obtained before any study-related activities take place. It is the responsibility of the Investigator or delegate to obtain written consent for each patient prior to performing any trial related procedures. The Investigator may delegate responsibility for obtaining written informed consent to members of the site research team who are appropriately trained in Good Clinical Practice (GCP). Delegation of responsibility must be indicated appropriately on the Site Signature and Delegation Log.

After a patient is approached and provided with a PIS, the patient must be given sufficient time to consider the verbal and written information before deciding to participate. The investigator or delegated individual should discuss the trial clearly and without bias and emphasize that the patient can refuse to participate or withdraw from the trial at any time without giving a reason, and that this would in no way influence their standard of care or relationship with the clinical team. The patient must be given the opportunity to ask questions which should be answered to their satisfaction.

If the patient decides to participate in the study they will be asked to sign and date the current approved version of the ICF. The person taking consent must then also sign and date the ICF. Only patients who are able and willing to give informed consent will be able to participate in the study.

A copy of the ICF should be given to the patient and a copy placed in the patient's notes along with the PIS. The original copy should be stored in the Investigator's Site File (ISF) at each site and a copy must also be sent to Sponsor.

Throughout the study, at each appointment, the patient should be given the opportunity to ask questions about the trial and ongoing consent should be confirmed verbally and recorded in the patient notes. The patient is free to withdraw from the study at any time and any new information relevant to their treatment should be shared with them in a timely manner.

6.3 Randomisation

After signing and dating the Informed Consent Form (ICF) the patient can be entered into the study and randomized. Randomization occurs via a database system at Regional Cancer Center (RCC) within Karolinska Institute, Sweden. In this system the inclusion and exclusion criteria will be automatically verified before patients are randomized. An outline of instructions for randomization can be found in Appendix 2. Full details of how to randomize a patient and access the randomization system will be provided by Karolinska Institute to local sites upon study commencement.

Upon randomization each patient will be allocated a unique Study Identification (ID) number which will be used to identify patients throughout the trial. This Study ID should be recorded on all study documents including the ICF and all CRF's and Questionnaires.

Patients will be randomized to either Group A (without Strattice™) or Group B (with Strattice™). Patients should be informed of their allocation promptly and a letter sent to their GP to inform them of their participation and treatment allocation.

6.4 Study settings and procedures

Surgeons performing IBR within the trial will be either plastic surgeons, or breast surgeons familiar with the ADM technique. Training instructions will be provided to reduce variations between centres. The surgical technique using ADM is outlined in Appendix 3.

The decision and treatment plan advocating mastectomy will be based on a multi-disciplinary conference (MDT), and the diagnosis of invasive or pre-invasive (in situ) breast cancer is made on triple assessment. If the patient wishes to undergo immediate implant based reconstruction, and meets the selection criteria for entering the study, they will be approached about the study.

Patients randomized to Group A (non-Strattice™) will be reconstructed with expander or anatomical gel implants without ADM, using complete muscle coverage. Group B (Strattice™) will be reconstructed with expander or anatomical gel implant using partial muscle coverage in conjunction with ADM. Randomization at enrollment is made with regard to the use of ADM or not, so both expander and direct-to-implant techniques will be utilized in both groups, depending on the quality of skin flaps at time of surgery.

All patients randomised to Group A, the standard (non-Strattice™) treatment will undergo a two stage post-mastectomy reconstruction using an expander prior to the implant being fitted. Patients randomised to Group B, the treatment arm using Acellular Dermal Matrix (Strattice™) will either undergo the same two-stage reconstruction but with the addition of the Strattice™ mesh, or a single-stage post-mastectomy direct-to-implant reconstruction which does not use an expander and in which the Strattice™ is fitted in the same procedure as the implant. The type of procedure used will be dependent on clinical factors such as the size and shape of the breasts and the surgeon will make this decision on a case-by-case basis. In cases where an expander is used the tissue expanders will be permanent expanders such as Becker 35 (Mentor©) or Style 150 (Allergan©), or anatomical gel implants such as Mentors© CPG series or from Arion Laboratories©.

6.5 Schedule of Events

Eligible patients will be approached about the study and given a Patient Information Sheet to read and will be given opportunity to discuss the trial with the Clinician or Research Nurse. If the patient decides to participate they will be asked to sign an Informed Consent Form. It is at this point that the patient enters the study and is randomized. A letter will be sent to the patient's GP informing them of their participation in the study and treatment allocation.

Patients will attend a Baseline visit at enrollment where they will undergo clinical assessments and complete a Baseline CRF and Questionnaires. This can be at the same time as signing of the ICF or can be at a separate visit.
After treatment allocation patients will be booked in for surgery as usual. All patients randomised to the standard treatment (non-Strattice™) will undergo a two stage post-mastectomy reconstruction using an expander prior to the implant being fitted. Patients randomised to the treatment arm using Acellular Dermal Matrix (with Strattice™) will either undergo the same two-stage reconstruction but with the addition of the Strattice™ mesh, or a single-stage post-mastectomy direct-to-implant reconstruction in which the Strattice™ is fitted in the same procedure as the implant. The type of procedure used will be dependent on clinical factors such as the size and shape of the breasts and the surgeon will make this decision on a case-by-case basis.

Therefore, patients receiving single stage direct-to-implant IBR will have a single surgical visit (Group B only) whilst those patients receiving two-stage IBR using an expander will have two surgical procedures in separate visits (all Group A patients and some Group B patients).

After surgery, patients in both groups will be discharged home with the drain in place (current standard of care) and the breast care nurses will remove the drain as per standard guidelines. The patients will be contacted by the research nurse after their discharge to assess whether they need to be brought back to hospital for an earlier review. Patients undergoing implant reconstruction with ADM are likely to have the drains left in situ for a longer duration to allow the ADM to adhere to the overlying skin

envelope and therefore ensure integration of the product. However, this should not lead to increased hospital stay and patients in both treatment arms will have the drained removed within 1-4 weeks

Patients will attend follow-up appointments at 1-4 weeks, and 3, 6, 12 and 24 months after their initial surgery (current standard care). During these visits the patient will undergo clinical assessments and will be asked to complete follow-up CRF's and questionnaires.
Where possible all study visits will coincide with routine clinical appointments. With the exception of the difference in surgical visits, all other trial visits and procedures are identical for both Group A and B.

6.6 Study completion

After the final 24 month follow-up visit patients will return to the standard care pathway and continue standard care follow-ups, which will be arranged at the time of discharge. Patients in the study are unlikely to require any further intervention once the research is finished. If there were any complications due to the use of Strattice™, the patient may need further treatment or an intervention to remove the Strattice™. In any such cases, patients would have direct access to clinical services as per standard care. If the complication developed during the study period, patients would not be discharged until it is suitably resolved, even if that period was to extend beyond the planned study period.

Patients will be able to contact the study team or clinical care team at any point during or after the study with any questions or concerns. Patients will also be given contact details for an independent patient group.

6.7 Study withdrawal

Patients are free to withdraw consent and end participation in this study at any time without having to give a reason and without current or future care being in any way affected. Patients should contact study team if they wish to end participation.

Participation in this study may be ended by the clinician or the sponsor without patient consent at any time in the following circumstances:

- Investigator thinks it is necessary for your health or safety;
- Patient has not followed study instructions;
- The sponsor has ended the study.

If a patient experiences disease progression or metastases they would receive whatever treatment is deemed necessary by the clinical care team, including further treatment or surgery, but this would not usually be a reason for study withdrawal. Any adjuvant therapy including radiotherapy, chemotherapy and immunotherapy are not an exclusion criteria would not be a reason for study withdrawal. If a patient experienced reconstructive failure or any complications which required the removal of Strattice™, patients would be withdrawn from the study and would be treated according to standard care.

If the patient decides to withdraw from the study, or is withdrawn, the study data collected prior to withdrawal may still be processed along with other data collected as part of the study but no future data will be collected. A withdrawal form in the CRF would need to be completed.

9 Study Data Collection

9.1 Case Report Forms (CRF's)

At each visit the investigator will obtain the required data to complete the Case Report Form (CRF). The CRF will record clinical and patient details such as medical history, demographics, breast measurements, and the presence of any pain or infection. There is a separate CRF for each visit, including at Baseline and all follow-up visits (1-4 weeks, 3, 6, 12 and 24 months). CRF's should be completed with the patient at their routine clinical appointment or by the clinical care team where appropriate. The baseline CRF should be completed prior to randomization.

9.2 Quality of Life Questionnaires

Patients will also be asked to complete standardized EORTC Quality of Life (QoL) questionnaires: C30 (generic), BR23 (breast cancer specific) and BRR26 (breast reconstruction specific) at specific time-points. EORTC QLQ-C30 and EORTC QLQ-BR23 are completed at baseline and 6, 12 and 24 month follow-up visits. EORTC QLQ-BRR26 is completed at 6, 12 and 24 month follow-up visits (see fig. 1). Patients should complete the questionnaires at the time of the clinic appointment where possible for data quality purposes. The baseline questionnaire should be completed prior to randomization. The table below shoes the study visits at which each questionnaire will be completed:

9.3 Photographs

Clinical photographs will be taken of the patient's breast(s) to document patient progress throughout the study, and evaluate the cosmetic and clinical results of the procedures. These will be taken at Baseline and at follow-up visits 3, 6, 12 and 24 months. The patient must consent for their photographs to be taken in order to be part of the study and this is clearly stated on the ICF. Aesthetic evaluation will be assessed using a 6-point scaled based scale for pre- and post-operative photographs.
To ensure data quality specific guidelines should be adhered to when taking photographs (see Appendix 4).

Figure 2 summarizes patient visits and data collection in study flow diagram.

Fig. 2 Study Flow chart

SCREENING
Patient information Sheet

ENROLLMENT
Consent Form
Randomisation

VISIT 0 - BASELINE
Physical assessment
Baseline CRF
Photographs
QoL Questionnaires (QLQ-C30, BR23)

SURGERY
Post-operative care

VISIT 1 – FOLLOW-UP
(1-4 weeks)
Removal of drain

VISIT 2 – FOLLOW-UP
(3 months)
Patient CRF
Photographs

VISIT 3 – FOLLOW-UP
(6 months)
Patient CRF
Photographs
Questionnaires (QLQ-C30, BR23, BRR26)

VISIT 4 – FOLLOW-UP
(12 months)
Patient CRF
Photographs
Questionnaires (QLQ-C30, BR23, BRR26)

VISIT 5 – FOLLOW-UP
(24 months)
Patient CRF
Photographs
Questionnaires (QLQ-C30, BR23, BRR26)

10 STUDY MANAGEMENT

The Lead Investigator at each site may delegate study tasks to suitably qualified members of the study team, which will be documented through a signed and dated Site Signature and Delegation Log (SSDL). However, the Lead Investigator remains ultimately responsible for study management and all study related activities.

All approved study documents should be correctly version controlled and printed or photocopied onto locally headed paper. Study data and documentation will be transferred via secure methods to Karolinska Institute, Sweden for analysis and review. Each site should keep local copies of all study related documents and data.

10.1 Data recording

Upon randomisation, each patient will be assigned a unique Study Identification (ID) consisting of a two-letter code corresponding to the site and ascending allocated numbers e.g. OX001 for the first patient randomized at Oxford. This will be recorded on a Screening Log with the birthdate and initials of each patient, and will be used on all study documents. In this way the data will be anonymized and the patient cannot be identified through the database or study documentation.

The investigators at each site must ensure that all data from patient visits are entered promptly on to the hardcopy Case Report Forms (CRF). Data should be verified against source records where appropriate. Entries on the CRF should be made in ballpoint pen, in black ink, and must be legible. Any errors should be crossed out with a single stroke, the correction inserted and the change initialed and dated. If necessary a file note should be included to detail the change and reason. If any information is not known, this must be c early indicated on the form. All missing and ambiguous data will be queried. All sections are to be completed before returning.

The lead investigator at each site must sign and date the final CRF page to attest to the accuracy and completeness of the data. The data from the CRF is then input at each site by a suitably delegated individual for analysis by the study team at Karolinska Institute. Hardcopies of the CRF’s will be transferred securely and stored at Karolinska Institute. Each site should also keep a copy of the CRF’s locally in the ISF.

10.2 Protocol deviations

Any protocol deviations should be reported immediately to the Sponsor.

10.3 Monitoring and auditing

At periods not exceeding 3 months, the Lead Investigators at Karolinska Institute will contact sites to discuss the progress of the trial. The study team will verify CRF data against source records for accuracy of data recording and collection, and confirm compliance with the protocol and the protection of patients' rights as detailed in the Declaration of Helsinki.

Additional on-site monitoring visits may be triggered, for example, by poor CRF return, poor data quality, low SAE reporting rates or an excessive number of withdrawals. Sites will be given sufficient notice and explanation. Investigators at each site must permit trial-related monitoring and audit and allow access to documentation as requested.

10.4 Archiving

It is the responsibility of the Lead Investigator at each site to ensure all essential trial documentation and source records at their site are securely stored and retained for at least 5 years after the end of the trial.

This is to ensure access to the details of intervention for the ongoing patient care as patients with breast cancer are routinely followed for 5 years and the information might have bearing on their ongoing clinical care. Also longer follow-up information might be relevant for the patients with ADM for clinical and aesthetic outcomes.

11 Safety Reporting

11.1 Adverse Events

An Adverse Event (AE) is defined as any untoward medical occurrence in a patient or clinical trial subject participating in the trial, which does not necessarily have a causal relationship with the treatment received.

At each visit after surgery the patient will be assessed regarding occurrence of any adverse events (AE), such as: capsular contracture, seroma, hematoma, infection, wound dehiscence/ wound breakdown and skin necrosis. Any adverse event will be recorded in the CRF Adverse Event (AE) page, along with the surgical area/site assessment, and any surgical intervention recorded.

11.2 Serious Adverse Events

A Serious Adverse Event (SAE) is defined as any untoward occurrence that:

- Results in death
- Is life-threatening
- Requires hospitalization or prolongation of existing hospitalization
- Results in persistent or significant disability or incapacity
- Consists of a congenital anomaly/ birth defect
- Other medically important event

9.3 Reporting procedure

The Chief Investigator at each site will be responsible for reporting and recording all adverse events. All observed or volunteered adverse events regardless of treatment group must be recorded on the Adverse Events page in the CRF. The lead investigator is responsible for assessing the causality and seriousness of all AE's and SAE's and must obtain adequate information to determine the outcome of the adverse event and assess whether it meets the criteria for classification as an SAE. Appendix 13.5 gives guidance on procedure and decision-making regarding Adverse Events.

All adverse events should be reported in a timely manner. Serious adverse events (SAE) should be immediately reported by the Chief Investigator to the Sponsor and LifeCell™ and a copy of any correspondence or information filed in ISF. The Sponsor is responsible for informing REC but this may be delegated to the CI. Any questions concerning adverse event reporting should be directed to the Chief Investigator in the first instance and then discussed with the Lead Investigators at Karolinska Institute, Sweden if necessary.

As stated in the Research Agreement with the Life Cell™ EMEA:

Coordinating investigators from each site shall report to Company any and all serious anticipated device related effects, device malfunctions, or protocol deviations within twenty-four (24) hours of the investigators becoming aware of such event. It is further agreed that coordinating investigators provide any such additional information as necessary to assist Company (Life Cell™) in complying with its reporting requirements as stated in the Research Agreement.

The lead investigators will monitor adverse events and safety reporting to ensure that any concerns are dealt with immediately. If there are any concerns about SAE's or the safety of the trial the study may be temporarily or permanently suspended. The REC will be notified immediately if a significant issue is identified during the course of the trial.

10. ETHICS

The clinical trial will be conducted according to the Declaration of Helsinki (Revised 2007) and local laws and regulations relevant to the performance of randomised trials of therapeutic procedures in the country of conduct. The trial will be conducted in accordance with the Research Governance Framework for Health and Social Care, the applicable UK Statutory Instruments, (which include the Data Protection Act 1998) and Good Clinical Practice (ICH GCP).

This study has been approved by the Central Ethical Review Board in Sweden (Registration number: 2012/1173-31/1), 22nd August 2012. The protocol will be submitted to and approved by the Research Ethics Committee (REC) in the UK prior to circulation.

Before any patients are enrolled into the trial, the Lead Investigator at each site is responsible for obtaining local R&D approval. It is the responsibility of the Principal Investigator to ensure that any subsequent amendments gain the necessary local approval.

Reporting

The CI shall submit once a year throughout the study, or on request, an Annual Progress report to the REC Committee, host organisation and Sponsor. In addition, an End of Study notification and final report will be submitted to the same parties.

11. Data and Publication policy

11.1 Data and Intellectual Property

The Lead Investigators retain the rights to data generated from this study and Intellectual Property (IP) rights will be agreed in Site Agreements. Lead Investigators share IP rights and all study data will be available for use – the commercial funder has no right to control data use and analysis. As stated in the Investigator Initiated Research Agreement with Life Cell™, that the Lead Investigators will have the “unfettered right to publish the outcomes and results of the Study, without interference from the Company”.

The results of this study may be communicated at scientific meetings and in publications if jointly agreed by all lead investigators. Publications must be agreed and discussed with the Lead Investigators, with authorship determined by mutual agreement. Any publications will be available in the public domain.

15. PUBLICATION POLICY

The publication policy should cover authorship, acknowledgements, and review procedures for scientific publications. If there is a department or institution policy, or agreement, the protocol can refer to it. Ensure that the publication policy stated here is consistent with any contract applicable to the study. Consider describing how study results may be disseminated to study participants.

The Investigators will be involved in reviewing drafts of the manuscripts, abstracts, press releases and any other publications arising from the study. Authors will acknowledge that the study was funded by LifeCell EMEA Ltd Authorship will be determined in accordance with the ICMJE guidelines and other contributors will be acknowledged.

11.2 Confidentiality and Data Protection

Potential participants would be initially identified by the Chief Investigator or clinical care team responsible for the routine care of the patient. Participants will be issued with a unique ID number when they enter the study and this will be used to identify the participants throughout the study. Only the direct care team and authorized individuals will have access to any identifiable data. All patient data will be anonymized and no

identifiable data will be on study documentation. The patients will not be identifiable when entered into the database.

A letter will be sent to the patient’s General Practitioner (G.P.) to inform them of their participation in the clinical research study - this is explained in the PIS and the patient must consent for this information to be shared on the ICF.

All clinical information will be securely stored and will only be accessible by authorized personnel for the purposes of this study. All members of the study team are employed substantially or have honorary contracts with OUH NHS Trust and are bound by confidentiality agreements. Representatives from the OUH NHS Trust hosting the trial and Sponsor may require access to medical records and patient data in order to conduct auditing or monitoring of the trial. This will be detailed on the consent form. Only authorized personnel with appropriate affiliation with the Trust will have access. Personal data recorded on all documents will be regarded as strictly confidential and will be handled and stored according to the Data Protection Act 1998. If the patient does not want their medical data to be accessed by a third party related to the study, they cannot participate in this study.

Photographic images will be taken by the clinician in a clinical setting - this would take place in standard care and would follow these guidelines. All photographs will be taken to exclude faces or any other identifying marks. Patients must consent for photographs to be taken for study purposes in order to participate in the study but can refuse consent for the photographs to be used for marketing or educational purposes. This is explained clearly in the PIS and Consent Form and can be discussed with the study team.

Any transfer of data or documents will be done in a secure manner using a secured, encrypted study specific equipment and database. At study end all documents will be securely archived for at least 5 years. The results of the study may be published for scientific purposes or used in future research but all information will be anonymized and unidentifiable.

12 Insurance and indemnity

The OUH NHS Trust has liability for clinical negligence that harms individuals toward whom they have a duty of care. NHS Indemnity covers NHS staff and medical academic staff with honorary contracts conducting the trial for potential liability in respect of negligent harm arising from the conduct of the study. The OUH NHS Trust is Sponsor and through the Sponsor, NHS indemnity is provided in respect of potential liability and negligent harm arising from study management.

13 Funding

LifeCell^{TM} EMEA Limited will be providing the Strattice^{TM} product free of charge for the purposes of this study. They will also provide study payments per patient to cover research related costs such as staffing and equipment. Details of these financial arrangements will be documented in the study contracts and site agreements. LifeCell^{TM} will provide funding for 30 patients recruited at Oxford - if the site recruits more patients they will continue to provide Strattice^{TM} product but will not provide any study payments.

14 REFERENCES

1. Becker S, Saint-Cyr M, Wong C, et al. AlloDerm versus DermaMatrix in immediate expander-based breast reconstruction: A preliminary comparison of complication profiles and material compliance. Plast Reconstr Surg. 2009;123:1–6; discussion 107–108.

2. 14. Liu AS, Kao HK, Reish RG, et al. Postoperative complications in prosthesis- based breast reconstruction using acellular dermal matrix. Plast Reconstr Surg. 2011;127:1755–1762

3. American Society of Plastic Surgeons. 2009 report of the 2008 statistics of plastic surgery statistics. Available at: http://www.plasticsurgery.org/Media/stats/2008- UScosmetic-reconstructive-plastic-surgery-minimally-invasivestatistics.

4. Atisha D, Alderman A, Lowery J, Kuhn L, Davis J, Wilkins E. Prospective analysis of long-term psychosocial outcomes in breast reconstruction: two-year post- operative results from the Michigan Breast Reconstruction Outcomes Study. Ann Surg 2008 Jun;247(6):1019e28.

5. Ho G, Nguyen TJ et al. A systematic review and meta-analysis of complications associated with acellular dermal matrix-assisted breast reconstruction. Ann Plast Surg. 2012 Apr;68(4):346-56.

6. Chun Y, Verma K, Rosen H, et al. Implant-based breast reconstruction using acellular dermal matrix and the risk of postoperative complications. Plastic Recon Surg 2010;125(2):429-36

7. Spear SL, Parikh PM, Reisin E, Menon NG. Acellular dermis assisted breast reconstruction. Aesthetic Plast Surg 2008;32: 418**e**25.

8. Zienowicz RJ, Karacaoglu E. Implant-based Breast Reconstruction with allograft. Plast Reconstr Surg 2007;120:373**e**81.

9. Sbitany H, Sandeen SN, Amalfi AN, Davenport MS, Langstein HN. Acellular dermis-assisted prosthetic breast reconstruction versus complete submuscular coverage: a head-to-head comparison of outcomes. Plast Reconstr Surg 2009;124:1735**e**40.

10. Breuing KH, Colwell AS. Immediate breast tissue expanderimplant reconstruction with inferolateral alloderm hammock and postoperative radiation: a preliminary report. Eplasty 2009;**9**:e16.

11. Breuing KH, Warren SM. Immediate bilateral breast reconstruction with implants and inferolateral AlloDerm slings. Ann Plast Surg 2005;55:232**e**9.

12. Salzberg CA. Nonexpansive immediate breast reconstruction using human acellular tissue matrix graft (AlloDerm). Ann Plast Surg 2006;57:1**e**5.

13. Topol BM, Dalton EF, Ponn T, Campbell CJ. Immediate singlestage breast reconstruction using implants and human acellular dermal tissue matrix with adjustment of the lower pole of the breast to reduce unwanted lift. Ann Plast Surg 2008;61:494**e**9.

14. Gamboa-Bobadilla GM. Implant breast reconstruction using acellular dermal matrix. Ann Plast Surg 2006;56:22**e**5.

15. Breuing KH, Colwell AS. Inferolateral AlloDerm hammock for implant coverage in breast reconstruction. Ann Plast Surg 2007;59:250**e**5.

16. Colwell AS, Damjanovic B, Zaherdi B, et al. Retrospective Review of 331 Consecutive Immediate Single-Stage Implant Reconstructions with Acellular Dermal Matrix: Indications, Complications, Trends, and Costs. Plastic Recon Surg 2011;128:1170.

17. Salzberg CA, Ashikari AY, et al. An 8-Year Experience of Direct-to-Implant Immediate Breast Reconstruction Using Human Accelular Dermal Matrix (Alloderm). Plastic Recon Surg 2011; 127: 514.

18. Jansen L, Macadam S. The use of Alloderm in postmastectomy alloplastic breast reconstruction: part II. A cost analysis. PLast Rec Surg 2011; 127 (6):2245-54

19. Komorowska-Timek E, Oberg KC, Timek TA, et al. The effect of AlloDerm envelopes on periprosthetic capsule formation with and without radiation. Plast Reconstr Surg. 2009;123:807–816.

20. Stump A, Holton 3rd LH, Connor J, Harper JR, Slezak S, Silverman RP. The use of acellular dermal matrix to prevent capsule formation around implants in a primate model. Plast Reconstr Surg 2009;124:82**e**91.

21. LifeCell Corp. AlloDerm, regenerative tissue matrix instructions for use. Available at: http://www.lifecell.com/downloads/ LC_Alloderm114_IFU_B_T4.pdf. Accessed December 12, 2009.

22. Weichman KE, Wilson SC et al. The use of acellular dermal matrix in immediate two-stage tissue expander breast reconstruction. Plast Reconstr Surg. 2012 May; 129(5): 1059-60.

23. Preminger B et al. The influence of Alloderm on expander dynamics and complications in the setting of immediate tissue/ implant reconstruction: a matched-cohort study. Ann of Plast Surg 2008;60(5):510-3.

24. Antony A, McCarthy C, Cordeiro P, et al. Acellular human dermis implantation in 153 immediate two-stage tissue expander breast reconstructions: determining the incidence and significant predictors of complications. Plast Reconstr Surg 2010;125(6):1606-14.

25. Connor J, et al. Retention of structural and biochemical integrity in a biologic mesh supports tissue remodeling in a primate abdominal wall model. Regen Med. 2009;4(2):185-195.

15 APPENDICES

15.1 Appendix (1): Definition of reoperations

As reoperation rates are a relatively objective measure of surgical intervention required to attain and maintain acceptable aesthetics in implant based reconstructions, it serves as a good primary endpoint for the study.

An unplanned reoperation in the study is defined as any subsequent surgical procedure including, but not limited to the following:

a. Reoperation caused by complications (e.g. implant removal or evacuation of hematoma or seroma) or due to cosmetic reasons, or implant exchange for other reasons (implant exchange from permanent expander to anatomical gel is considered as an unplanned reoperation).
b. Capsulectomy/ capsulotomy
c. Any secondary autologous reconstruction
d. Lipofilling

15.2 Appendix (2): Instructions for Randomisation

<u>Randomising a patient</u>

Randomisation service website: https://data.dynareg.se/adm

Contact details for login: christofer@lagerros.se

1. Log on to: https://www2.onkc.ks.se/adm

2. Enter the patient ID in the format of the two-letter organizational code and patient number in ascending order e.g. KS001 for the first patient to be randomized.

3. Select Fill in new form > Randomization

4. Complete the questions and click ‘Save’.

5. Check the "Perform randomization" box before saving

6. Once a patient is randomized, you cannot change the data

15.3 Appendix (3): Surgical technique using ADM (Strattice™) in IBR [11]

Strattice™ is used in conjunction with the implant working as an extension of the PMM, allowing for vascular ingrowth and as such, serving as a scaffold for the development of tissue formation.

1. Preoperatively: Breast meridian, IMF, and perimeter of breast mound are marked with permanent pen. The base perimeter and volume of breast is used as guide to selection of implant size. Skin incision (NSM, SSM) is based on tumor location.
2. Following completion of mastectomy, viability of the skin flaps must be judiciously evaluated, i.e. with temporary sizer in place, to determine whether expander or permanent implant should be used. If any concern regarding perfusion of skin flaps, an expander should be used.
3. The inferolateral origin of PMM is elevated off the chest wall along the IMF, using electrocautery, leaving pectoralis minor, serratus anterior, and rectus abdominis muscles untouched. A small portion of the most inferomedial attachment of pectoralis major is released, but leaving the sternal origin mostly undisturbed.
4. **Before being used, the Strattice™ piece is soaked and placed in saline at room temperature for at least 2 minutes, and rinsed extensively several times to wash off any preservative fluid.**
5. The Strattice™ piece is taken from saline bath and placed directly into the wound, so as not to contact the operating field or the patients skin. The matrix is preferably only handled by one surgeon and sewn along the level of the previously marked IMF using interrupted 2-0 PDS© preferably. To recreate the lateral aspect of the breast mound, The ADM piece is curved laterally and cranially and sutured to the Serratus anterior muscle, which is not elevated off the chest. If necessary a second piece of ADM can be sewn to reach full coverage.
6. The mastectomy skin flaps are tailored to the breast mound, leaving as little dead space as possible between the dermal matrix and the skin flaps, to minimize seroma formation.

7. **Two closed-suction drains are placed.** One along the IMF, between subcutaneous tissue and Strattice™, and one along the superior perimeter of the breast/ axilla in the implant pocket.
8. **Drains are removed when less than 30 ml fluid / drain/ 24h, but should stay a maxium of 10 days postoperatively**

Special note 1

If a permanent gel implant is chosen, it is of crucial importance that the size of the implant does not put any strain on the skin envelope upon wound closure, so as not to compromise the skin circulation and ultimately, jeopardize the postoperative healing. The skin flaps should be draped over the breast mound without any tension and closed in layers. Also, **if skin flaps are aggressively trimmed during mastectomy leaving several patches of only dermis, using a tissue expander is highly recommended.**

ANTIBIOTICS REGIMEN:

A 4-dose regime of antibiotics (*2g Cloxacillin iv)* will be administered the first 24 hours, with the first dose given in conjunction with the surgery in theatre. Extended use of antibiotics might be considered if drains are not removed before discharge from hospital.

Special note 2

In both groups, any seroma formation after drain removal should be treated as early as possible with percutaneous drainage. **Preferably with ultrasound guidance and a dose of prophylactic antibiotics given post-operatively.**

15.4 Appendix (4): Standard procedures for taking photographs

Photographs should be taken in front of a black background with 4 different views according to the templates below. All jewellery should be removed and any distinguishing marks such as tattoos should be concealed.

Position 1: front, navel visible, hands on hips	Position 2: front, arms stretched above head, shoulders relaxed and parallel.
Position 3: rotate body to the right 45 degrees, right arm is not visible.	Positions 4: rotate body to the left 45 degrees, left arm is not visible.

15.5 Appendix(5): Defining a surgical infection

i. Signs suggesting infection, such as redness and/ or edema of skin, should be confirmed by elevated levels of C-Reactive Protein (CRP) to qualify as an infection.

ii. In case of wound dehiscence/ wound breakdown and/ or oozing, bacterial culture is taken.

iii. Also to be recorded is whether there is skin dehiscence only, muscle/ implant exposure and/ or skin flap necrosis. **If signs of skin flap necrosis, any expander in place should be deflated.**

iv. In case of localized redness/ erythema of the skin overlying ADM, without elevated levels of CRP and LPK, the event is to be regarded as local inflammation, and should be treated with Non Steroid Anti-Inflammatory (NSAID) drugs.

v. Any seroma formation in the ADM group should be treated aggressively with ultra sound guided percutaneous drainage and patient put on prophylactic antibiotics for 7 days.

vi. If surgical intervention with removal of implant is decided, both implant and ADM should be removed. If regarded as an infection, three levels of treatment can be defined:

1. Oral antibiotics
2. Intravenous antibiotics with patient treated at ward in hospital
3. Infection requiring implant removal
