# Supplementary material for: Effect of Immediate Implant-Based Breast Reconstruction After Mastectomy With and Without Acellular Dermal Matrix Among Women With Breast Cancer: A Randomized Clinical Trial
Source: JAMA Netw Open. 2021 Oct 1;4(10):e2127806. doi: 10.1001/jamanetworkopen.2021.27806 (PMC8486981; doi:10.1001/jamanetworkopen.2021.27806)
Supplement: Supplement 2. — eMethods. eTable. Patient Reported Scores at 24 Months for the Breast Reconstruction-Specific EORTC-BRR26 Questionnaire [file jamanetwopen-e2127806-s002.pdf]

Supplemental Online Content

Lohmander F, Lagergren J, Johansson H, Roy PG, Brandberg Y, Frisell J. Effect of immediate implant-based breast reconstruction after mastectomy with and without acellular dermal matrix among women with breast cancer: a randomized clinical trial. *JAMA Netw Open.* 2021;4(10):e2127806. doi:10.1001/jamanetworkopen.2021.27806

eMethods.

eTable. Patient Reported Scores at 24 Months for the Breast Reconstruction-Specific EORTC-BRR26 Questionnaire

This supplemental material has been provided by the authors to give readers additional information about their work.

© 2021 Lohmander F et al. *JAMA Network Open.*

eMethods

Procedures

Diagnosis of invasive or pre-invasive (in situ) breast cancer was based on triple assessment (clinical examination, imaging and core biopsy), as per routine protocol in the respective breast units. Decisions and treatment-plans recommending mastectomy were discussed at local multidisciplinary meetings.

The decision of using a definitive gel implant or utilizing a tissue expander was based on clinical judgement after assessing the viability of the skin flaps, and whether the sub-muscular pocket was large enough and of adequate quality, to accommodate a fixed-volume implant. In both groups a temporary sizer was used when appropriate, to optimize the match between the skin envelope and implant volume. Two drains were placed, one in the implant cavity and one subcutaneously. For the ADM group the drains remained in place until the output was <30 ml per 24 h for two days in a row, for a maximum of 14 days. For the control group, drains were removed when output was <50 ml per 24 h. All patients received one dose of prophylactic antibiotic (1g Cloxacillin i.v.) before start of surgery, followed by three doses within the next 24 h. No further antibiotics were given. All patients were instructed to wear a post-surgical bra for two weeks after surgery. Tissue expansion was started in the out-patient clinic around three weeks post-op according to standard practice. All patients received implants made by Mentor© (Santa Barbara, California, USA) or Allergan™ (Dublin, Ireland).

After signing informed consent, participants were invited to complete the generic QLQ-C30 and breast cancer specific QLQ-BR23 questionnaires in the out-patient setting, or if preferred, returning the questionnaires by pre-paid mail to the coordinating research nurse. The questionnaires were administered at baseline (pre-randomization) at the clinic. Follow-up questionnaires were administered by mail, including instructions for completion and a return envelope at three time points: six, 12- and 24-month post reconstruction. A reminder was sent from the research nurse within 2-3 weeks.

Questionnaires

EORTC QLQC30 consists of 30 items comprising five function scales: physical (PF), emotional (EF), social (SF), role (RF), and cognitive (CF); and three symptom scales: fatigue (FA), nausea/vomiting (NV), and pain (PA). Six single items are also included: dyspnea (DY), insomnia (SL), appetite loss (AP), constipation (CO), diarrhoea (DI), and financial difficulties (FI). The final two items assess global health and overall quality of life. Most items are responded to on a four-point scale ranging from 1 (not at all) to 4 (very much). The two items assessing global health and overall quality of life are responded to in seven categories ranging from 1 (very poor) to 7 (excellent).

EORTC QLQ-BR23, comprises 23 questions, constituting five multi-item scales assessing disease symptoms such as arm and breast symptoms, side-effects of treatment (surgery, chemotherapy, radiotherapy and endocrine treatment), body image and sexual functioning.[19] In addition, sexual enjoyment, hair loss and future perspectives are measured by single items. The response format is the same as for the core questionnaire.

EORTC QLQ-BRR26 consists of 26 items (with scores ranging from 1 ('Not at all') to 4 ('A lot') constituting seven scales: disease treatment/surgery related symptoms, problems finding a well-fitting bra, sexuality, cosmetic outcome breast, cosmetic outcome donor site, satisfaction with reconstructed nipple, and problems with losing the nipple. The questionnaire was validated and tested for reliability in a set of breast cancer patients after breast reconstruction. The Swedish version was a part of the development of this questionnaire.[20, 21]

© 2021 Lohmander F et al. *JAMA Network Open.*

eTable. Patient reported scores at 24 months for the breast reconstruction-specific EORTC-BRR26 questionnaire

	IBBR with ADM (n = 64)[*]	IBBR without ADM (n = 65)[*]	Mean difference[†]		P-value
**EORTC QLQ-BRR26**					
Domain					
Disease treatment/ surgery related symptoms	7 (15)	10 (16)	-3 (-11, 6)		0.442
Problems finding a well-fitting bra	19 (25)	31 (32)	-13 (-28, 3)[M]		0.038
Sexuality	34 (26)	37 (10)	-1 (-16, 13)		0.797
Cosmetic outcome breast	68 (23)	60 (24)	8 (-5, 20)[S]		0.111
Cosmetic outcome donor site	n.o.	n.o.	n.o.		
Satisfaction with reconstructed nipple	57 (23)	49 (27)	11 (-6, 28)[M]		0.112
Problems with losing the nipple	33 (33)	43 (24)	-5 (-30, 20)[S]		0.602

[*]Values are mean (range 0-100), with standard deviations in parenthesis. [†] A higher score indicates higher satisfaction for ´Satisfaction with reconstructed nipple and ´Cosmetic outcome of breast`, for all other domains a lower score indicates higher satisfaction. [S] = Small clinical difference, [M] = Moderate clinical difference (Osoba 1998). ADM, acellular dermal matrix; n.o., no observations.

© 2021 Lohmander F et al. *JAMA Network Open.*
