# Supplementary material for: Effect of Immediate Implant-Based Breast Reconstruction After Mastectomy With and Without Acellular Dermal Matrix Among Women With Breast Cancer: A Randomized Clinical Trial
Source: JAMA Netw Open. 2021 Oct 1;4(10):e2127806. doi: 10.1001/jamanetworkopen.2021.27806 (PMC8486981; doi:10.1001/jamanetworkopen.2021.27806)
Supplement: Supplement 3. — Data Sharing Statement [file jamanetwopen-e2127806-s003.pdf]

Lohmander F, Lagergren J, Johansson H, Roy PG, Brandberg Y, Frisell J. Effect of immediate implant-based breast reconstruction after mastectomy with and without acellular dermal matrix among women with breast cancer. *JAMA Netw Open.* 2021;4(10):e2127806. doi:10.1001/jamanetworkopen.2021.27806

Data Sharing Statement

Data
Data available: No

Additional Information
Explanation for why data not available: Data is can be accessed upon request. Data in paper format
